# Plasma cadmium and zinc and their interrelationship in adult Nigerians: potential health implications

**DOI:** 10.1515/intox-2015-0012

**Published:** 2015-06

**Authors:** Emmanuel Ike Ugwuja, Lawrence Ulu Ogbonnaya, Henry Uro-Chukwu, Johnson Akuma Obuna, Emeka Ogiji, Simon Uchenna Ezenkwa

**Affiliations:** 1 Department of Chemical Pathology, Faculty of Medicine, Ebonyi State University, P.M.B. 053 Abakaliki, Nigeria; 2 Department of Biochemistry, Faculty of Sciences, Ebonyi State University, P.M.B. 053 Abakaliki, and Nigeria; 3 Department of Community Medicine, Ebonyi State University, P.M.B. 053 Abakaliki, Nigeria; 4 Department of Social Mobilisation and Disease Control, National Obstetric Fistula Centre, Abakaliki, Nigeria; 5 Department of Obstetrics and Gynaecology, Faculty of Medicine, Ebonyi State University, P.M.B. 053 Abakaliki, Nigeria; 6 Department of Pharmacology and Therapeutic, Faculty of Medicine, Ebonyi State University, P.M.B. 053 Abakaliki, Nigeria.

**Keywords:** cadmium, zinc, anthropogenic activities, pollutant, heavy metal toxicity

## Abstract

Zinc (an essential trace element) and cadmium (a ubiquitous environmental pollutant with acclaimed toxicity) have been found to occur together in nature, with reported antagonism between the two elements. The present study aimed at determination of plasma levels of zinc (Zn) and cadmium (Cd) and their interrelationship in adult Nigerians. The series comprised adults (n=443) aged ≥18 yrs (mean ± SD 38.4±13.7 yrs), consisting of 117 males, 184 non-pregnant and 140 pregnant females. Sociodemographic data were collected by questionnaire while anthropometrics were determined using standard methods. Plasma Cd and Zn were determined by using an atomic absorption spectrophotometer. The mean plasma zinc and cadmium were 94.7±18.1 μg/dl and 0.150±0.548 μg/dl, respectively. Age, sex, pregnancy, and parity had no effect on either plasma Zn or Cd. Although educational level had no effect on plasma Zn, it had a significant effect on Cd; subjects possessing either secondary or tertiary education had significantly lower plasma Cd than subjects without formal education. Moreover, there seemed to be an inverse relationship between Cd and Zn, but this was not statistically significant (r=–0.089; *p*=0.061). Although plasma Zn was not related to BMI (r=0.037; *p*=0.432), Cd was significantly negatively correlated with BMI (r=–0.124; *p*=0.009). It may be concluded that adult Nigerians in Ebonyi State have elevated plasma levels of Cd, with apparent impact on the levels of plasma Zn. This has important public health implications considering the essential roles of Zn in the protection of Cd mediated adverse health effects. While food diversification is recommended to improve plasma Zn, efforts should be made to reduce exposure to Cd to mitigate partially its possible adverse effects.

## Introduction

Cadmium, a non-essential toxic metal in group IIB of the periodic table has been identified as a ubiquitous environmental pollutant with acclaimed toxicity at extremely low concentration. The toxicity of cadmium affects the bone, liver, renal, reproductive (Obianime & Roberts, 2009) and respiratory systems, and DNA repair mechanism (Anetor, 2012), ultimately precipitating diseases, such as hypertension, emphysema, cancer (Julin *et al*., 2012; Kheradmand *et al*., 2013; Joseph, 2009), to mention but a few. Sources of Cd in the environment include anthropogenic activities and electronic wastes as Cd has been widely used in industry, such as electroplating and galvanizing, mining and processing of metals, in pigments of paints and plastics, components of batteries and electronic circuits in computers and cell phones, and of printers’ ink (Anetor, 2012). Human exposure occurs through contaminated food, water and cigarette smoking (Anetor, 2012; Orisakwe *et al*., 2012).

Although populations occupationally exposed to or resident in areas contaminated with Cd are exposed to higher Cd concentrations than the general public, Cd has become an insidious component of food chains even in unexposed industrialized countries (WHO, 1992; Anetor, 2012).

Mechanisms of Cd toxicity include modification of biomolecles, modulation of DNA repairs and genotoxic consequences (Anetor, 2012), antagonism of zinc, induction of oxidative stress, and impairment of p53 protein involved in suppression of cancer.

Zinc, an essential trace element has been found to occur together with Cd in nature. Metallothionine, a zinc metalloprotein, was reported to protect against Cd-induced tissue damage. Pre-treatment with Zn was found to be protective against Cd toxicity, with significant decrease in serum Zn (Jamai *et al*., 2010). Cd binds to metallothionine more tightly than does Zn (Klaassen *et al*., 1999). Moreover, intestinal absorption of Cd appears to be influenced by micronutrients including Zn (Kippler *et al*., 2009).

Although previous studies in Ebonyi State have revealed that concentrations of heavy metals, including Cd and Zn, are high in soil, food, and water (Onwuchekwa *et al*., 2009; Ogbodo, 2013; Edeogu *et al*., 2007) and that heavy metal uptake by crops growing on contaminated soil is a potential hazard to human health due to transmission in the food chain (Staessen *et al*., 1994; Ekpo *et al*., 2014), there are no records of plasma levels of Cd or Zn among the residents of the state considering the reported antagonism between the two elements. The present study therefore aimed at determining the plasma levels of Cd and Zn as well as the relationship between the two elements in adult residents of the state. The outcome may have relevance in designing mitigating measures against Cd toxicity and in preventing adverse health consequences associated with Cd exposure.

## Materials and methods

### Study area

Ebonyi State is located on longitude 8° E and latitude 6° N with a moderate relief of between 125 m and 245 m above sea level. The vegetation characteristics are those of the tropical rain forest with an average annual rainfall of about 1,600 mm and average atmospheric temperature of about 30 °C. The State has 3 Senatorial Districts (Ebonyi South, Central and North), 13 Local Government Areas (LGAs) and 215 political wards (EBSIEC, 2011).

### Methods

This study was conducted between April 2013 and June 2014. It was part of a larger study intended to correlate some toxic metal contents of foods and waters in Ebonyi State with their levels in blood of residents and associated biochemical consequences. Briefly, by random sampling, ten (10) wards were selected from each of the 13 Local Government Areas. Altogether 130 wards were involved in the study. Inclusion criteria were: being resident in the selected wards for ≥1 year, aged ≥18 years, no history of trace element supplementation in the last six months and being apparently healthy. Exclusion criteria included history of chronic diseases, including liver and renal diseases, diabetes, malignancy, sickle cell anemia, or seropositivity to human immunodeficiency virus (HIV). First, advocacy for the study was made. The leaders of the different churches and trade unions were consulted and informed of the rationale for the study. Some churches allowed members of the study team to address the congregation on the need for the study, while others relayed the message as explained to them with members of the study team on hand to ascertain the clarity of the information passed. The village heads and councils were notified and news was passed to the involved subjects during their town meetings. At all these meetings eligible ward members were encouraged to come out at a designated central location on a set date for enrolment into the study with assurance that every eligible volunteer will be allowed to participate at no cost. To facilitate coverage, a center was mapped out and volunteers were asked to assemble at the center on the appointed date.

Those in attendance at each study center received information from the team physicians on environmental pollution, with specific reference to toxic metals (arsenic, cadmium and lead) and to zinc. The rationale and study objectives were explained to the subjects and consent requested. Volunteers gave written consent to participate in the study after which their socio-demographic characteristics were collected using a structured questionnaire administered by one of the study team members in the native language of the participants. Then medical examination of each participant was carried out. Height and weight measurements were taken of the subjects in light clothing without shoes, caps or head tie, using a standard calibrated meter rule affixed to a wall perpendicular to a flat smooth surface floor, while the body weight was measured using a digital weighing scale (Seca, Harmburg, Germany). The body mass index (BMI) was calculated by dividing the weight of the participant (kg) by the square of height (m^2^). Blood samples for the determination of cadmium and zinc concentrations were collected in trace element-free lithium heparin bottles (Becton-Dickinson, Rutherford, NJ, USA). The samples were transported in ice packs to the laboratory, where plasma was separated and frozen before analysis. Plasma cadmium and zinc concentrations were determined using an atomic absorption spectrophotometer (Bulk Scientific, Model AVG 210). Certified Cd and Zn reference solutions (obtained from Sigma-Aldrich Co. LLC, USA) for atomic spectrometry were used as controls. Intra-individual variability was minimized by ensuring that samples were analyzed in duplicates and the mean taken as the final reading, while inter-individual variation was eliminated by ensuring that the same person analyzed all samples.

### Ethical consideration

The Ethics and Research Committee of the Federal Teaching Hospital, Abakaliki, Ebonyi State, approved the protocol of the study. The approval was given on the agreement that patient anonymity must be maintained, good laboratory practice/quality control ensured, and that every finding would be treated with utmost confidentiality and for the purpose of this research only. All work was performed according to the international guidelines for human experimentation in clinical research (WMA, 2000). Subjects with a serious medical condition were referred to the nearest hospital or to the Federal Teaching Hospital, Abakaliki for further assessment and management.

### Data handling

Data were analyzed by using Statistical Package for Social Sciences (SPSS^®^) for Windows^®^ version 16 (SPSS Inc., Chicago, IL, USA). Values were expressed as mean ± standard deviation. Multiple comparisons were done using one-way analysis of variance (one-way ANOVA) with level of significance set at *p*<0.05. Correlation was determined by Pearson correlation analysis.

## Results

Table 1 shows the general characteristics of the subjects. The mean age of the subjects was 38.4±13.7 years (range 18–83 yrs), mean BMI: 24.4±4.2 kg/m^2^ (range 15.8–44.8 kg/m^2^), mean plasma Cd: 0.150±0.548 μg/dl (range 0.001–4.874 μg/dl), and mean plasma Zn: 94.7±18.1 μg/dl (range 47.6–200.5 μg/dl).

**Table 1:** General characteristics of the subjects.

Parameters	n	Min.	Max.	Mean	SD
Age (yrs)	443	18	83	38.4	13.7
BMI (kg/m^2^)	443	15.8	44.8	24.4	4.2
Plasma Cd (μg/dl)	443	0.001	4.874	0.150	0.548
Plasma Zn (μg/dl)	443	47.6	200.5	94.7	18.1

**BMI:** Body mass index

Table 2 shows that significantly higher plasma Cd levels were observed among adult residents of Ezza South > Ivo > Ohaukwu > Ebonyi > Izzi > Ezza North in comparison to inhabitants of other LGAs. For plasma zinc, the lowest value was observed in residents of Ohaukwu and Ezza North, with residents of Ohaozara < Izzi < Afikpo North < Afikpo South < Ikwo < Onicha having significantly lower values than inhabitants of other LGAs.

**Table 2:** Plasma Cd and Zn according to local government areas.

	LGAs	BMI	Cadmium (μg/dl)	Zinc (μg/dl)
Ebonyi North	Abakaliki (40)	26.5±5.6^a^	0.006±0.003^a^	98.9±10.1^a^
	Ebonyi (40)	24.6±3.2^b^	0.059±0.024^a^	96.3±16.2^a^
	Izzi (38)	26.4±5.0^a^	0.045±0.026^a^	92.2±21.4^b^
	Ohaukwu (33)	24.3±4.7^b^	0.485±0.034^b^	86.6±11.3^c^
Ebonyi Central	Ezza South (43)	22.0±2.5^c^	0.761±1.051^c^	99.3±19.3^a^
	Ishielu (40)	23.4±3.6^c^	0.004±0.022^a^	109.1±16.7^d^
	Ezza North (37)	23.7±3.8^c^	0.018±0.011^d^	86.6±15.7^c^
	Ikwo (33)	24.9±5.4^b^	0.007±0.002^a^	93.2±11.9^b^
Ebonyi South	Onicha (22)	23.5±4.6^c^	0.009±0.002^a^	93.8±22.8^b^
	Afikpo North (35)	24.0±4.3^b^	0.007±0.002^a^	92.3±15.7^b^
	Afikpo South (25)	23.8±4.1^c^	0.007±0.002^a^	92.8±15.7^b^
	Ivo (21)	24.9±2.0^b^	0.550±1.080^b^	95.4±26.3^b^
	Ohaozara (34)	25.6±2.3^a^	0.008±0.002^a^	90.217.0^b^

**BMI:** Body mass index; **LGAs:** Local Government Areas

Values with different superscript are statistically different (*p*<0.05)

Body mass index (BMI) was found to be higher among residents of Ebonyi North Senatorial zone in comparison to other zones, with the highest values among residents of Abakaliki and Izzi LGAs. However, while comparable BMI values were observed among residents of Ebonyi Central and Ebonyi South, the lowest BMI value was recorded among residents of Ezza South.

In males, non-pregnant females and pregnant females, there was no significant difference in plasma concentrations of Cd and Zn (Table 3). However the females in general (non-pregnant and pregnant) had a significantly higher BMI than their male counterparts (Table 3).

**Table 3:** Comparison of BMI, plasma Cd and Zn among male, nonpregnant female and pregnant female.

Parameters	Male (n=117)	Non-pregnant female (n=184)	Pregnant female (n=140)
BMI (kg/m^2^)	23.3±3.5^a^	24.7±4.8^b^	25.1±3.9^b^
Cadmium (μg/dl)	0.116±0.54	0.208±0.65	0.104±0.38
Zinc (μg/dl)	93.4±18.0	94.9±19.7	95.6±16.1

**BMI:** Body mass index

Values with different superscript are statistically different (*p*<0.05)

Table 4 shows the body mass index (BMI), plasma Cd and Zn in relation to age groups of subjects. The age group 31–40 years had a significantly higher BMI in comparison to other age groups. Although the age group ≤30 years had a higher BMI than the age groups 41–50 and >50 years, the difference was not statistically significant. Plasma Cd seemed to be higher (*p*>0.05) among older age groups in comparison to the younger age groups, while zinc concentrations were comparable among the age groups.

**Table 4:** Body mass index, plasma cadmium and zinc in relation to age-groups.

Parameters	Age groups (yrs)				
	≤30 (n=151)	31-40 (n=127)	41-50 (n=72)	>50 (n=93)
BMI (kg/m^2^)	24.1±3.5^a^	25.9±4.9^b^	23.7±3.6a^c^	23.5±4.3bc^d^
Cadmium (μg/dl)	0.096±0.04	0.151±0.50	0.207±0.71	0.194±0.69
Zinc (μg/dl)	95.4±16.6	94.4±16.8	95.8±20.2	93.2±20.7

**BMI:** Body mass index

Values with different superscript are statistically different (*p*<0.05).

Table 5 shows that BMI was observed to be significantly (*p*<0.05) higher among the higher parity groups (3, 4, & >4) in comparison to the lower parity groups (0, 1 and 2). However, parity did not have any significant effect on the plasma levels of either Cd or Zn as comparable values were observed among the groups.

**Table 5:** Plasma Cd and Zn in relation to parity.

Parity	Age (yrs)	BMI (kg/m^2^)	Cadmium (μg/dl)	Zinc (μg/dl)
0 (49)	23.3±5.5	23.1±2.^a^	0.085±0.36	97.9±17.1
1 (22)	25.1±5.8	25.2±4.7^a^	0.022±0.02	89.3±10.8
2 (35)	29.8±8.5	24.1±3.8^a^	0.285±0.84	94.9±16.4
3 (35)	32.8±8.1	25.1±3.5^b^	0.253±0.67	94.3±16.3
4 (51)	37.0±10.5	25.0±4.8^b^	0.177±0.55	96.4±20.3
>4 (127)	42.110.9	25.6±5.1^b^	0.182±0.58	94.7±19.5

**BMI:** Body mass index

Values with different superscript are statistically different (*p*<0.05)

Table 6 shows the effect of educational status on BMI, plasma Cd and Zn. Although educational status did not exert any significant effect on BMI and plasma Zn, subjects with either secondary or tertiary education had significantly lower plasma Cd levels in comparison to those with no formal education.

**Table 6:** BMI, cadmium and zinc levels in relation to educational level (number of subjects in parenthesis).

Educational level	BMI (kg/m^2^)	Cadmium (μg/dl)	Zinc (μg/dl)
None (84)	23.6±5.8	0.255±0.76	95.1±23.92
Primary (159)	23.8±4.0	0.196±0.64	93.5±16.1
Secondary (158)	25.3±4.4	0.084±0.35*	96.2±17.3
Tertiary (42)	25.5±5.1	0.015±0.02**	92.8±15.0

**BMI:** Body mass index

* Significant difference from none and secondary (*p*=0.021)

** Significant difference from none and tertiary (*p*=0.020)

**Figure 1:**
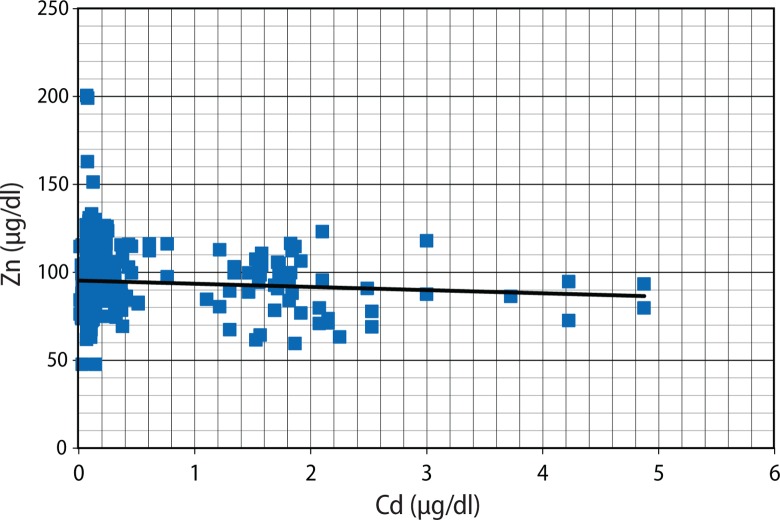
Scatter plots of Plasma Zn against Cd.

**Figure 2:**
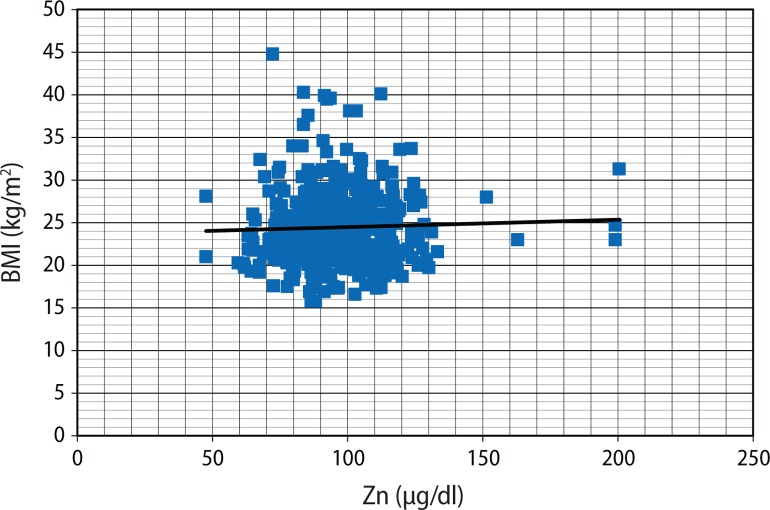
Scatter plots of BMI (kg/m^2^) against plasma Zn (μg/dl).

**Figure 3:**
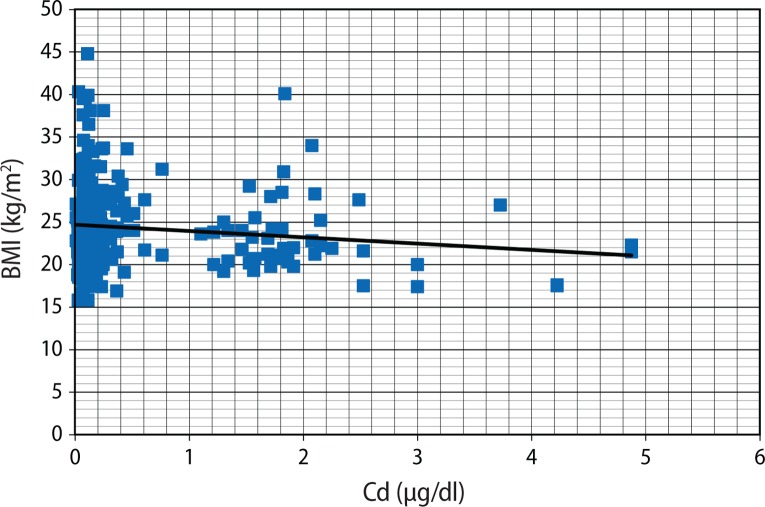
Scatter plots of BMI (kg/m^2^) against plasma Cd (μg/dl).

Figure 1 seems to present an inverse relationship between Cd and Zn, but this was not statistically significant (r=–0.089; *p*=0.061). Although plasma Zn was not related to BMI (r=0.037; *p*=0.432) (Figure 2), Cd was negatively correlated with BMI (r=–0.124; *p*=0.009) (Figure 3).

## Discussion

The ranges of plasma Zn (47.6–200.5 μg/dl) and Cd (0.001–4.874 μg/dl) observed in the present study revealed wide disparity in the distribution of these elements in the state. This is consistent with previous studies suggesting that geographical variations and environmental exposure contribute to blood Cd levels (Watanabe *et al*., 2004) along with industrial exposure, diet and ethnicity. Although we did not encounter any previous study that would assess plasma levels of Zn and Cd among the residents of the state, high levels of these elements have been observed in soil, water, foods and grinding stones in different studies in the state (Edeogu *et al*., 2007; Onwuchekwa *ets al*., 2009; Ehiri *et al*., 2010; Ogbodo, 2013). It may thus be argued that the disparity in Cd and Zn levels observed among the residents of the state in the present study may in part be attributed to consumption of food and drinking water contaminated with these elements. This assertion stems from the report of Jarrup and Akesson (2009), which revealed that food grown on Cd-contaminated farmland was the main source of Cd exposure in non-smoking populations. Omaka *et al*. (2008) reported earlier that one of the major routes of exposure to metals in Ebonyi State was the background geochemistry as the state is highly mineralized with abundant deposits of Pb, Zn, *etc.* Cd was also found to be highly dispersed in the environment with significant contributions from anthropogenic pursuits, such as agricultural activities, fuel combustion, *etc.* (Omaka *et al*., 2011).

Significantly higher plasma Cd levels observed among adult residents of Ezza South > Ivo > Ohaukwu > Ebonyi > Izzi > Ezza North and the corresponding lower values for Zn is in agreement with the earlier hypothesis of Levander and Cheng (1980) and Mills (1981) stating that overabundance of one trace element can interfere with the level and metabolic utilization of another element present in normal or marginal concentrations. Cd and Zn occur usually together in nature because of their similar chemical properties and suggestive evidence of an interaction between Zn and Cd exposure has been reported (van Wijngaarden *et al*., 2008).

These findings have important public health implications for the adult residents of the state. While Cd is toxic to a number of organs and tissues in the body, Zn is an essential trace element involved in many crucial metabolic processes including cell differentiation, the immune system, hormone secretion, reproductive functions, to mention but a few. For instance, residents of a village where soil levels of Cd were high were reported to have raised blood pressure and biochemical findings, including hypertension and evidence suggestive of renal damage (Carruthers & Smith, 1979). The implication is that adult residents of the state are at risk of cardiovascular diseases, including raised blood pressure, hypertension and renal disorders. Although the present study did not assess renal functions or document blood pressure in the residents, a previous study in an agrarian rural community in the state documented hypertension prevalence of 23.2%, with age, consumption of red meat, body mass index (BMI), and the number of children in the family to be associated with hypertension (Ugwuja *et al*., 2015). Several mechanisms have been proposed through which Cd could raise blood pressure. These include poisoning of renal tubules, which may result in chronic renal disease and ultimately cause raised blood pressure, sympathetic over-activity through replacement of other divalent cation in enzymes that inactivate catecholamines, catechol-o-methyl-transferase and monoamine oxidase. Thus by acting as an inhibitor of these enzymes and natural antidepressant, Cd may elevate blood pressure and mood simultaneously, as well as toxicity to the testes, which may lead to low testosterone production and be associated with cardiovascular disease (Carruthers & Smith, 1979). It might be inferred that the urinary abnormalities earlier reported among young adults in the state (Ugwuja & Ugwu, 2008) may be partly attributed to the toxic effects of Cd. Further, exposure to environmental Cd was associated with reduced human male sperm concentration and motility (Benoff *et al*., 2009). Thus the high prevalence (70%) of low sperm counts and semen parameters reported earlier among male partners of women in the infertility clinic in Abakaliki (Ugwuja *et al*., 2008) may be connected with the impact of elevated blood Cd in the subjects. The reproductive toxicity of Cd in subjects of our study may be accentuated by low Zn levels as Zn plays an important role in reproduction. As an antioxidant nutrient, Zn maintains antioxidant defence against reactive species, which have been implicated in reproductive failures. Again, Zn was shown to be protective against Cd toxicity (Jamai *et al*., 2010).

Additionally, decreased Zn levels have been suggested to be permissive to DNA damage, which can lead to mutation, an important phenomenon in many cancers (Clayson, 2000). Zinc deficiency was found to disrupt P_53_ tumor suppressor protein (Ho *et al*., 2003). Loss of P_53_-mediated apoptosis, secondary to altered Cd:Zn ratio, has been suggested to be a rate limiting step in prostate tissue tumorigenessis (Anetor *et al*., 2008). Therefore residents of the state may also be at additional risk of various types of cancer, particularly prostate cancer, due to the observed Cd and Zn levels in the present study as environmental and dietary Cd exposure may play a role in the development of prostate cancer (Julin *et al*., 2012). Mechanisms implicated in Cd induction of carcinogenesis include: oxidative stress, inhibition of DNA repair, stimulation of cell proliferation, blockage of apoptosis and epigenic mechanisms (Hartwig, 2010).

The reason for the lack of significant difference in plasma Cd concentration between males and females in the present study is not known, yet dietary intake of Cd was reported to be higher in men than in women while Cd retention was found to be higher in the latter than in the former (Watanabe *et al*., 2004). According to the authors, the difference may be attributed in part to increased Cd absorption through the intestinal divalent metal transporter II (DMT II) when iron status is low, *e.g.* during menstruation (Vahter *et al*., 2007), as the difference in blood Cd between sexes was found to become normalized after menopause (Baecklund *et al*., 1999). However, the significantly higher BMI in females (both pregnant and non-pregnant) appears to be due to accumulation of fats during pregnancy. Parous women may retain more of their pregnancy weight, a condition that has been linked to long-time obesity (Schauberger *et al*., 1992).

The lack of a significant effect of age on plasma Cd and Zn in the present study is quite intriguing as the study showed that blood Cd but not Zn concentration may not become apparent until subjects are older than 40 years (Oldereid *et al*., 1993). A reasonable proportion (165/443; 37.2%) of our subjects were older than 40 years and a relationship is expected between age and plasma Cd. Thus the lack of relationship observed may be partly attributed to the fact that the majority of the subjects were within range of their 40ies as Cd has been identified as a cumulative toxicant.

The reason for the lack of the effect of parity on plasma Cd and Zn observed in the present study need to be further explored as this runs contrary to the earlier concept of increased DMT II-mediated Cd absorption during menstruation. This is in contrast to lower plasma Zn earlier reported in parous women in comparison to other parity groups (Ugwuja *et al*., 2010). It is also in contrast to the findings of Neggers *et al*. (1996) and Hanachi *et al*. (2014) in pregnant women where plasma zinc was related to parity. The disparity in their finding and that of the present study may be related to differences in the subjects. While their study was done in pregnant women, the present study involved both sexes, including pregnant and non-pregnant women. Educational status as the single index of socioeconomic status that has an effect on plasma Cd showed significantly lower plasma Cd in subjects with either secondary or tertiary education in comparison to those without formal education. Although we did not come across any study that evaluated the relationship between educational status and plasma Cd, elevated blood Pb levels were previously reported among women from low SES as represented by women without formal education and those whose living accommodation was a single room, with farming as occupation (Ugwuja *et al*., 2013). It has been argued that women from low SES are more likely to have high blood levels of heavy metals, including Cd because of increased environmental, occupational and dietary exposure, exemplified by farming and procurement of their foodstuffs from outdoor markets characterized by low levels of hygiene. For instance, high Pb levels were reported in some foods sold in outdoor markets in Nigeria (Nnorom *et al*. 2005; Maduabuchi *et al*. 2006; Adekunle *et al*. 2009). The same can be said of Cd as elevated Cd has been reported in foods and vegetables cultivated in the state (Edeogu *et al*., 2007). Again, subjects from higher socioeconomic classes may be in a better position in terms of nutrition and health education and are thus able to protect themselves against exposure to heavy metals. This is quite understandable as educational attainment has been established as a social variable that often displays the largest socioeconomic influence (Andersen & Mortensen, 2006) because it affects both income and occupation. Educated individuals are also more likely to understand public-health messages (Ugwuja *et al*., 2007) and to maintain higher personal hygiene than less-educated individuals. They may also have better access to adequate medical care and nutrition than their uneducated counterparts.

The lack of a significant relationship between Cd and Zn observed in the present study is in contrast with the inverse relationship observed elsewhere (Aneto *et al*., 2008). The reason may be partly attributed to the subjects. While in the study mentioned above, the subjects were strictly smokers in comparison to non-smokers, our analysis included both smokers and non-smokers. However the inverse relationship between BMI and plasma Cd in the present study calls for cautious interpretation as this implies that increased plasma Cd may be associated with reduced BMI or vice versa. Although the mechanism by which elevated plasma Cd affects BMI is obscure, it may be speculated that by inducing Zn deficiency increased plasma Cd may affect the plasma volume. In pregnant women for instance, it has been suggested that maternal micronutrient status plays a role in the regulation of body size and mediates plasma volume expansion (Neggers *et al*., 2003).

In light of the present study it may be concluded that adult Nigerians in Ebonyi State have elevated plasma levels of Cd, which appear to have an impact on the levels of plasma Zn. This has important public health implications considering the essential roles of Zn in the protection of Cd mediated adverse health effects. In the absence of a well defined relationship between the two elements, diversification of diet is recommended to mitigate the possible adverse effects of Cd among the residents of the state.
